# Trophic Dynamics and Feeding Ecology of Skipjack Tuna (*Katsuwonus pelamis*) off Eastern and Western Taiwan

**DOI:** 10.3390/molecules27031073

**Published:** 2022-02-05

**Authors:** Yun-Chen Chang, Wei-Chuan Chiang, Daniel J. Madigan, Fu-Yuan Tsai, Ching-Lung Chiang, Hung-Hung Hsu, Shiow-Mei Lin, Mei-Ying Zhuang, Ching-Ting Sun, Lu-Chi Chen, Sheng-Ping Wang

**Affiliations:** 1 Marine Fisheries Division, Fisheries Research Institute, Jhongjheng District, Keelung 202, Taiwan; ycchang01@mail.tfrin.gov.tw; 2 Penghu Marine Biology Research Center, Fisheries Research Institute, Magong City 880, Taiwan; hmlin@mail.tfrin.gov.tw (S.-M.L.); myzhuang@mail.tfrin.gov.tw (M.-Y.Z.); cjsun@mail.tfrin.gov.tw (C.-T.S.); lcchen@mail.tfrin.gov.tw (L.-C.C.); 3 Eastern Marine Biology Research Center, Fisheries Research Institute, Chenggong 961, Taiwan; a0911734080@gmail.com (F.-Y.T.); hhung.18@gmail.com (H.-H.H.); 4 Department of Integrative Biology, University of Windsor, Windsor, ON N9B 3P4, Canada; daniel.madigan@stonybrook.edu; 5 Department of Environmental Biology and Fishery Science, National Taiwan Ocean University, Jhongjheng District, Keelung 202, Taiwan; dragon579513@hotmail.com (C.-L.C.); wsp@mail.ntou.edu.tw (S.-P.W.)

**Keywords:** pelagic, predator, diet, stable isotope analysis, Bayesian mixing models, Northwest Pacific

## Abstract

The skipjack tuna (*Katsuwonus pelamis*) is a mesopredator fish species with seasonal abundance in waters off Taiwan. Regional ecological and life-history information has been historically lacking for this species. In recent years, stable isotope analysis (SIA) of carbon and nitrogen has been used to assess predator feeding ecology and broader ecosystem trophic dynamics. This study evaluated comparative skipjack feeding ecology in distinct regions off Taiwan, combining traditional stomach content analysis with SIA of individuals off western (*n* = 43; 2020) and eastern (*n* = 347; 2012–2014 and *n* = 167; 2020) Taiwan. The stomach content analysis showed the most important prey to be ponyfish (*Photopectoralis bindus*) in western Taiwan and epipelagic squids (*Myopsina* spp.) and carangids (*Decapterus macrosoma*;) in eastern Taiwan from 2012 to 2014 and epipelagic carangids (*Decapterus* spp.) and flying fishes (*Cheilopogon* spp.) in eastern Taiwan in 2020, suggesting that the skipjack tuna is a generalist predator across regions. In contrast, time-integrated diet estimates from Bayesian mixing models indicated the importance of cephalopods and crustaceans as prey, potentially demonstrating more mesopelagic feeding in less productive waters during skipjack migrations outside the study regions. Skipjack off western Taiwan had a slightly higher estimated trophic position than in the waters off eastern Taiwan, potentially driven by the varying nutrient-driven pelagic food web structures. Skipjack SI values increased with body size off eastern Taiwan but not in western waters, suggesting that opportunistic predation can still result in different predator–prey size dynamics between regions.

## 1. Introduction

The skipjack tuna (*Katsuwonus pelamis*) is a mid-trophic level, highly migratory fish species that is distributed throughout tropical and subtropical waters, with seasonal abundance in waters off Taiwan. Skipjack tuna are the third highest harvested fish globally over the past 9 years, with annual catches of up to 3.2 million tons, and is an important source of dietary protein [1]. The western and central Pacific Ocean (WCPO) is the main fishing ground for tuna purse-seine fisheries, with skipjack comprising the highest proportion of catch followed by yellowfin tuna (*Thunnus albacares*) [2]. The catch of skipjack tuna has increased from 1 million tons to 1.8 million tons in the western and central Pacific Ocean region in the past 20 years [3], showing the increasing commercial demand for skipjack tuna.

Adult skipjack generally migrate with the sea surface isotherm of ~15 °C, though this ranges from 14.7 to 30 °C. However, the thermal range of juvenile fish is relatively narrow, with low tolerance for temperatures <25 °C [4]. Skipjack depth use generally ranges from the sea surface to 260 m during the day and is predominantly constrained to the surface mixed layer at night. In eastern Taiwan, electronically tagged, immature skipjack tuna show deeper diving during daytime, performing repetitive bounce-diving behavior to depths between 50–310 m, with vertical use of the water column most likely linked to foraging [5].

Historically, skipjack trophic dynamics and feeding ecology have not been well described in western North Pacific pelagic systems. Based on more recent studies of pelagic food web structure [6], skipjack tuna are likely important mediators in the transfer of energy from primary producers to apex predators such as billfish, sharks, and larger tunas. There have been recent, related studies using stable isotope analysis (SIA) of apex predators in the Northwest Pacific pelagic ecosystem describing trophic changes with size in sailfish (*Istiophorus platypterus*) [7] and the feeding ecology and trophic position of blue marlin (*Makaira nigricans*) and black marlin (*Istiompax indica*) [8,9] off eastern Taiwan, and reconstructions of the pelagic food web structure in the Kuroshio Current have shown that skipjack tuna are likely a mesopredator species in the western North Pacific. Prior feeding studies of juvenile skipjack tuna in the tropical western Pacific have shown the dietary importance of fish larvae, cephalopods, and pelagic crustaceans, demonstrating generalist feeding [10]. Larger skipjack are more piscivorous in the Central Pacific, and inshore skipjack consume more reef-originating organisms than those from offshore areas [11], further indicating region-specific, opportunistic predation. By better describing the regional trophic dynamics and feeding ecology of skipjack, the role of this species across and within sub-regions of pelagic ecosystems can be better understood.

SIA of carbon (δ^13^C) and nitrogen (δ^15^N) has been used to reconstruct food webs and assign consumer trophic positions [12,13]. Prior to SIA, the most common method to analyze predator–prey relationships has been stomach content analysis (SCA) [14]. While providing high resolution, species-specific diet data, SCA has specific limitations. The different digestion rates of different prey species can cause the relative importance of some prey species to be underestimated [15]. As SCA generally represents a few days of feeding, it cannot fully show the predator’s diet over seasonal timeframes [16]. Prey importance estimates from SCA can also be biased by digestion rates, which vary with water temperature and prey size [17]. In contrast, based on isotopic turnover rates in mesopredator tissues, SIA integrates dietary patterns over longer timeframes, with the isotopic composition of fish muscle tissue reflecting the feeding patterns of prior months [18] to over one year [19]. Combining SCA and SIA can thus provide a more comprehensive picture of the ecological niche and primary forage base for tunas and other pelagic predators [20].

SIA has been increasingly used to describe feeding habits and spatiotemporal diet changes [21] to better understand the relationships between predator and prey species. δ^13^C and δ^15^N have been shown to vary geographically depending on the local environment’s dynamics; for example, in North Pacific embayments, δ^13^C and δ^15^N values increased with temperature, and δ^15^N values varied with the ambient nitrate concentration [22]. In contrast to minimal trophic fractionation of ^13^C, δ^15^N values increase with higher trophic positions, due to the observed successive ^15^N fractionation of ~2.5–3.5‰ with each trophic step [23]. As such, δ^15^N values have typically been used to assess predator trophic levels within a given system. Prior studies have used SIA to explore whether pressure from fisheries and/or long-term climate change may affect the transfer of marine nutrient energy through pelagic food webs [24,25,26]. From phytoplanktonic baselines through trophic transfers in pelagic food chains, the environmental conditions of oceanographically different waters can be passed from primary producers to top consumers [27]. Currently, SIA continues to be widely used to describe feeding habits [28] and the changes in diet across time and space [21], including changing predator–prey relationships.

Both δ^13^C and δ^15^N propagate through regional pelagic food webs, reflecting the productivity dynamics and trophic structure that vary by region. For example, SCA and SIA of skipjack tuna in different waters in the Northwest Atlantic showed region-specific diet differences, and that skipjack from different regions could be distinguished by SI values [12]. The different regional availability of prey may also affect predator movements, as has been shown by the response of North Atlantic salmon to the varying local abundance of prey species [29]. As other environmental factors also affect the growth and aggregation of predators, including temperature, prey type, and nutrient availability [30,31,32], migratory skipjack tuna will likely orient to environments most suitable for growth and survival. Underpinning these dynamics are regional assessments of skipjack and the surrounding ecosystem trophic dynamics, which have been relatively few. Finally, mesopredators shape ecosystems in both pelagic ecosystems [33] and more broadly [34]. Due to these ecological roles, gaining insight into mesopredator diets and their associated shifts in response to spatiotemporal variation is crucial for predicting ecosystem responses to fluctuating abundances.

In this study, SIA was used to investigate the comparative trophic dynamics of skipjack tuna off western and eastern Taiwan, investigating diet composition, ontogenetic trophic shifts, and regional diet differences. Isotopic data were used in Bayesian mixing models to construct trophic relationships between predator and prey species in these systems. Combining SCA and SIA here provides insights into the regionally and temporally variable trophic ecology of skipjack tuna, providing the opportunity to better understand the role these mesopredators play in sub-regions of the Northwest Pacific pelagic ecosystem.

## 2. Materials and Methods

### 2.1. Site Description, Data Collection, and Sampling

**Figure 1:**
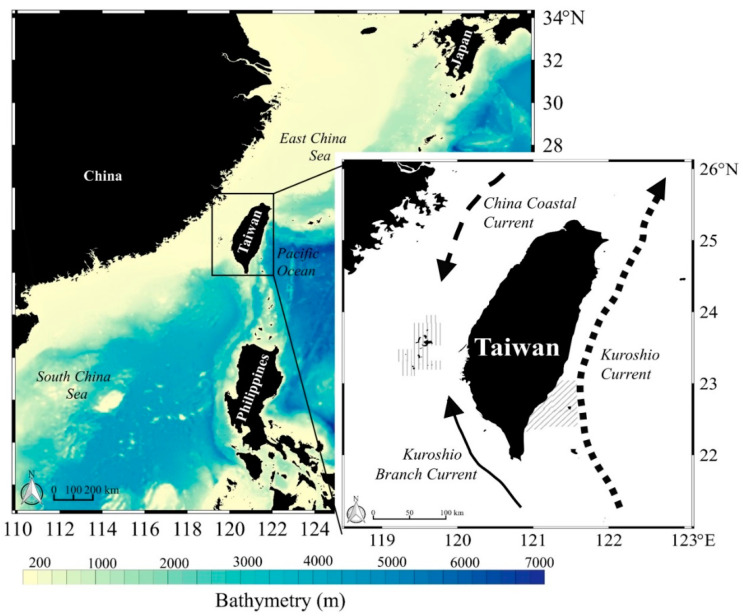
Sampling areas for skipjack tuna (Katsuwonus pelamis) and prey in western (2020) and eastern (2012–2014 and 2020) waters off Taiwan. Individual skipjack were collected from fish markets, with muscle tissue and stomachs retained for diet analyses. Sampling areas in western and eastern Taiwanese waters are shown (vertical and diagonal lines). Black dashed arrows show the China Coastal Current and Kuroshio Branch Current, and the solid arrow indicates the Kuroshio Current (redrawn from [35]).

Individual skipjack tuna were collected from western (Penghu landing) Taiwan in 2020 and eastern (Taitung landing) Taiwan in 2012–2014 and 2020 (Figure 1). In eastern Taiwan, samples were collected from the oligotrophic Kuroshio current (regionally high surface temperatures and salinity), which flows past eastern Taiwan throughout the year and drives topographic upwelling. The western Taiwan region is influenced by three major ocean currents: the China Coastal Current (lower temperature and salinity), and the Kuroshio Branch and South China Sea Currents (higher temperature and salinity). The seasonality of these ocean current dynamics off western Taiwan produces a more variable oceanographic environment than off eastern Taiwan, where the Kuroshio Current is more stable.

Skipjack tuna were caught by torch light net, set net, and trolling line fishery by fishermen. A total of 43 individuals were caught in western Taiwan, 347 specimens in eastern Taiwan throughout 2012–2014, and 167 specimens in eastern Taiwan in 2020 for subsequent analyses. The fork length (cm) and round mass (kg) were recorded for each fish. Dorsal white muscle samples were collected at the laboratory and then frozen at −80 °C.

### 2.2. Stomach Content Analysis

After skipjack tuna were collected from the fish market, their stomachs were immediately removed from each individual fish and stored in a 10% formalin solution for preservation. Before prey species identification, the stomach contents were water-rinsed for 24 h. The stomach contents were identified by morphological characters, using vertebral counts and structure for more intact items and hard parts (squid beaks, otoliths) for the digested material. When multiple otoliths were present from a single species, the total number of that species was estimated to be half of *n* otoliths. The prey species, number, and mass were recorded. The SCA calculations included stomach content weight index (SCWI), frequency of occurrence (FO%), percent by number (N%), percent by mass (W%), and Index of Relative Importance (IRI). These were calculated according to the following: SCWI is the proportion of the stomach mass to fish mass; FO% is the frequency of occurrence for each prey item relative to the total number of stomachs containing prey; N% is the proportion of the number of a given prey item to the total number of all prey items; W% is the proportion of the total mass of a given prey item to the total mass of all prey items [14]; and IRI provides a comprehensive metric of the importance of a given prey item, according to the equation [36]:

IRI = (W% + N%) × FO%(1)

### 2.3. Stable Isotope Analysis

White muscle samples were first rinsed with 10% HCl to remove inorganic carbonates and we washed the samples with DI water to remove any residue before analysis. The samples were then dried in a drying oven at 60 °C for 48 h. After drying, the white muscle samples were ground into powder and 0.6–0.8 mg was packaged into a tin capsule for analysis [37]. The samples were combusted in the elemental analyser (Flash EA-2000, Thermo-Finnigan; www.thermoscientific.com (accessed on 24 September 2021)) to produce CO_2_ and N_2_, which flowed through a gas chromatography (GC) column for separation. The separated gas then flowed through Conflo IV into a mass spectrometer (Thermo Delta V advantage). All the samples were analysed at the Global Change Research Center, National Taiwan University.

The stable isotope ratios of carbon and nitrogen are expressed in δ-notation, which is expressed by the following equation:

(2) $$\delta X=(\frac{R_{\mathrm{sample}}}{R_{\mathrm{standard}}}-1)\;\times \;1000$$

where X is ^13^C or ^15^N and R is the ratio of heavy isotope to light isotope (^13^C/^12^C or ^15^N/^14^N) [13]. The isotopic values are expressed in units of thousandths or per mille (‰).

We used δ^15^N values to estimate individual skipjack trophic position (TP) using the following [23]:

(3) $$\mathrm{TP}=\lambda +\frac{\delta^{15}N_{\mathrm{secondary}\;\mathrm{consumer}}-{\;\delta }^{15}N_{\mathrm{base}}}{\Delta_{n}}$$

where λ is the trophic position of the selected baseline organism, δ^15^N_secondary consumer_ is the skipjack δ^15^N value, δ^15^N_base_ is the δ^15^N value of the selected baseline (here, zooplankton or squid; see below), and Δ^15^N is the trophic discrimination factor (TDF) or change in δ^15^N between consumer and prey. We chose different baseline organisms for the west and east Taiwan study regions, based on the availability of region-appropriate baseline data. For our local baseline in western Taiwanese waters, we used SI values of mixed zooplankton, considering this to be the most appropriate baseline for pelagic food webs. We used an δ^15^N value of 6.3‰ for this mixed zooplankton (Jinn-Shing Weng, unpublished data) with an assumed trophic position of 2. We recognize that this approach cannot account for regional and temporal fluctuations in zooplankton δ^15^N values but consider these limitations inherent to this approach and still allow for useful comparisons of TP across mesopredators, which integrate isotopic baselines over periods of months to years. The baselines for eastern Taiwan were taken from previous SIA studies in the region [38], using a squid δ^15^N value of 8‰ [38], with an assumed trophic position of 3. For our applied TDF value, we chose the tuna-specific value derived from the long-term captivity of the Pacific bluefin tuna (*T. orientalis*) of 1.9‰ [19].

The prey species for SIA were selected based on their presence and importance in SCA (high IRI values) and the local availability of prey species. For western Taiwan, prey items were collected from fish markets and analyzed according to the above, and some prey SI data were taken from previous SIA studies [39]. The prey SI data for eastern Taiwan were taken from previous SIA studies in the region [8,39,40]. For all skipjack and prey tissues, C/N ratios were used to evaluate the need for lipid extraction due to lipid effects on δ^13^C values. Most values were ~3.3 and none were above 3.5, the recommended threshold for lipid correction or extraction [41,42]. As such, we report empirical values of δ^13^C.

### 2.4. Data Analysis

SI values for skipjack tuna were divided by sampling region (east and west), and further grouped into size classes depending on the size range in each region: (west: class I, <39.9 cm; class II, 40–49.9 cm; class III, >50 cm; east: class I, <49.9 cm; class II, 50–59.9 cm; class III, >60 cm). We used the Bayesian mixing model MixSIAR [43] to estimate the contribution of each prey type to each size class in both regions. We used TDF values of Δ^13^C = 1.8 ± 0.3‰ and Δ^15^N = 1.9 ± 0.4‰ in Bayesian mixing models, based on values reported for Pacific bluefin tuna and related species [19]. While recognizing that mixing models are sensitive to TDF selection [44] and can be affected by diet [45], TDF values have been shown to vary substantially around model fits [45]. Therefore, we found empirical lab-derived values from similarly sized tuna, which are also supported by another North Pacific mesopredator teleost [46], to be most appropriate.

For prey groupings, accepting that individual species could not be used due to input limitations in mixing models, we classified the main prey species of skipjack tuna (from SCA) into taxonomically and ecologically relevant groups (Table 1). Groupings prioritized prey of observed importance while also integrating other potentially important prey, given the temporal limitations of SCA. Recognizing mesopelagic feeding in other regions and the possibility that skipjack feed on other prey items outside the study region, we included pelagic cephalopods and crustaceans in mixing models using values from the literature. Due to the possibility of high skipjack feeding on other prey across seasons not sampled for SCA, we used uninformed priors. For model inputs, Markov Chain Monte Carlo (MCMC) was set to test length, error structure was the ‘resid’ process, and we used both Gelman–Rubin and Geweke diagnostics to test for model convergence. All mixing models were run for 10^5^ iterations.

Comparisons were made for SI values of skipjack across regions, time period, and size. We used T-TEST to test for differences in SI values between body size, regions, and years, and used linear regression to test for differences in δ^13^C and δ^15^N values across fork length.

## 3. Results

### 3.1. Size Class

A total of 43 individuals were collected for analysis in western Taiwanese waters. In eastern Taiwan, a total of 514 individuals were collected, 347 specimens in 2012–2014 and 167 in 2020 (Figure 1). The fork length (FL; cm) of the skipjack tuna were different across regions and timeframes, likely due to the migratory pathways and differences in cohort mixing: size in western Taiwanese waters ranged from 34–56 cm (45.0 ± 4.2 cm), in eastern Taiwanese waters in 2012–2014 from 35–80 cm (47.2 ± 8.5 cm), and in eastern Taiwanese waters in 2020 from 41–72 cm (54.1 ± 9.2 cm). Individual skipjack mass ranged from (west) 0.8–3.6 kg (1.9 ± 0.5 kg), (east; 2012–2014) 0.2–10.0 kg (2.1 ± 1.4 kg), and (east; 2020) 1.2–8.0 kg (3.4 ± 1.9 kg). Individual skipjack from eastern Taiwanese waters were significantly larger than those in western Taiwanese waters (*t*-test, *p* < 0.05).

### 3.2. Stomach Content Analysis

#### 3.2.1. Regional Comparisons

**Figure 2:**
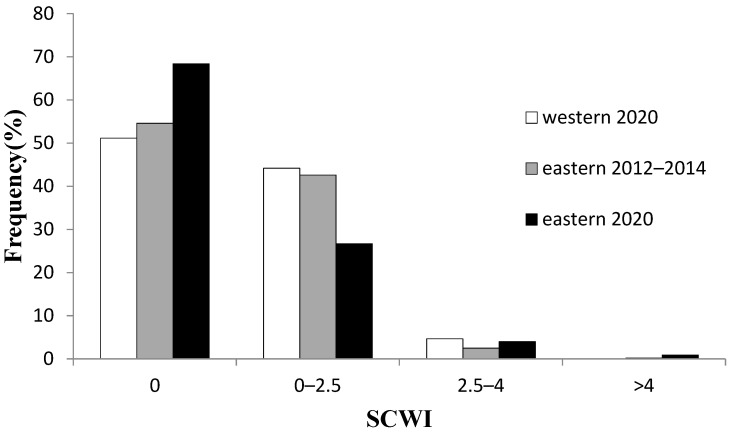
Stomach content weight index (SCWI) frequency distribution for skipjack tuna collected in western and eastern Taiwanese waters. A score of 0 = empty, 0–2.5 = low fullness, 2.5–4 = moderate fullness, and >4 = completely full.

In western Taiwanese waters, more than half of stomachs were empty (SCWI = 0; 51.2%). In eastern Taiwanese waters, 54.6% (2012–2014) to 68.4% (2020) of stomachs were empty (Figure 2). There was no significant difference in the percentage of empty stomachs between western and eastern regions. However, ingested prey was different in the western and eastern Taiwanese waters (Table 2, Table 3 and Table 4). Based on calculated IRI, the main prey of skipjack tuna in western Taiwan were ponyfish (*Leiognathus bindus*; IRI 3282.9), followed by round herring (*Spratelloides gracilis*; IRI 277.6) and lanternfish (*Benthosema pterotum*; IRI 96.8). In eastern Taiwan, the main prey were squids (*Myopsida* spp.; IRI 1265.9), carangids (*Decapterus* spp.; IRI 2054.3), and flying fishes (*Cheilopogon* spp.; IRI 609.0).

#### 3.2.2. Temporal Comparisons in Eastern Taiwan

The importance of prey items in eastern Taiwan varied between the two sampling periods. In 2012–2014, the main prey were squids (*Myopsida* spp.; IRI 1265.9), followed by carangids (*Decapterus macrosoma*; IRI 412.34) and sand eels (*Ammodytidae* spp.; IRI 347.74). In 2020, the main prey were carangids (*Decapterus* spp.; IRI 2054.3), followed by flying fishes (*Cheilopogon* spp.; IRI 609.0) and squids (*Myopsida* spp.; IRI 442.68).

### 3.3. Stable Isotope Analysis

#### 3.3.1. Regional Comparisons

Skipjack δ^15^N values ranged from 8.2 to 12.3‰ in western Taiwan and from 6.3 to 12.6‰ in eastern Taiwan. The δ^13^C values ranged from −18.9 to −16.9‰ in western Taiwan and from −18.9 to −15.5‰ in eastern Taiwan. There were significant differences (*t*-test, *p* < 0.05) in mean (±SD) δ^15^N values in western (11.2 ± 0.7‰) and eastern (9.8 ± 1.2‰) Taiwan (Table 5). There were significant differences (*t*-test, *p* < 0.05) in trophic position in western (4.5) and eastern (3.9) Taiwan. There was no difference (*t*-test, *p* > 0.05) in mean (± SD) δ^13^C values in western (−18.0 ± 0.4‰) and eastern (−17.9 ± 0.4‰) Taiwan (Table 5).

**Figure 3:**
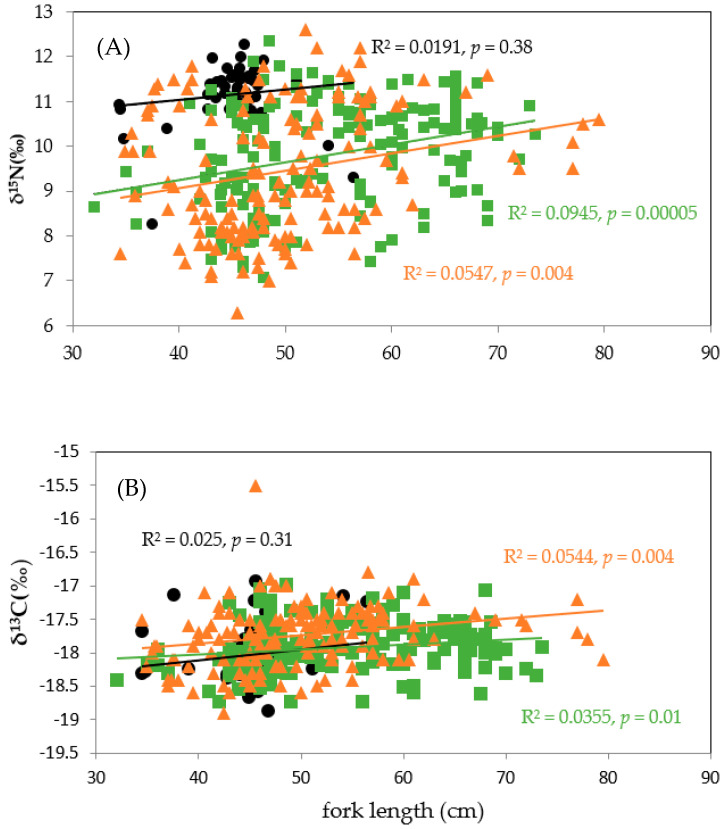
Relationships between size (fork length, cm) and muscle (**A**) δ^15^N values and (**B**) δ^13^C values for skipjack tuna (*Katsuwonus pelamis*) off western Taiwan (*n* = 43); eastern Taiwan 2012–2014 (*n* = 149); and eastern Taiwan 2020 (*n* = 167). Symbols represent (●) western Taiwan (*n* = 43), (▲) eastern Taiwan 2012–2014 (*n* = 149), and (■) eastern Taiwan 2020 (*n* = 167). Trendlines shown are for linear regressions, with associated equations, R^2^, and *p* values shown.

There was no significant relationship (linear correlation, *p* > 0.05) between fork length and δ^15^N or δ^13^C values in western Taiwan (Figure 3A,B). There was a positively correlated relationship (linear correlation, *p* < 0.05) between fork length and δ^15^N and δ^13^C values in eastern Taiwan (Figure 3A,B).

**Figure 4:**
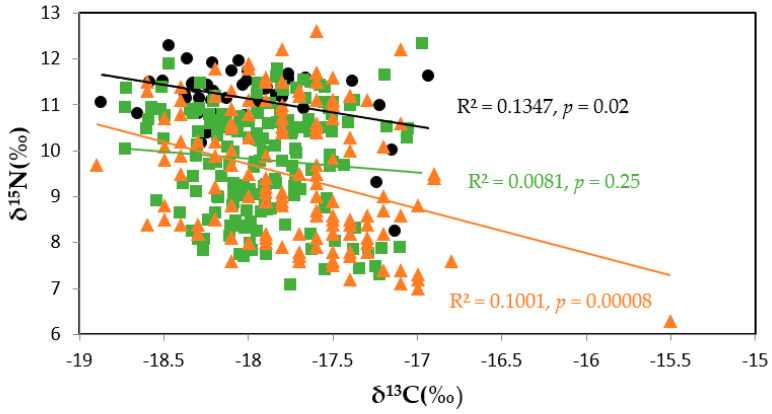
The relationships between δ^15^N and δ^13^C values of skipjack tuna (*Katsuwonus pelamis*) off western Taiwan (*n* = 43), eastern Taiwan 2012–2014 (*n* = 149), and eastern Taiwan 2020 (*n* = 167). Symbols represent (●) western Taiwan (*n* = 43), (▲) eastern Taiwan 2012–2014 (*n* = 149), and (■) eastern Taiwan 2020 (*n* = 167). Trendlines shown are for linear regressions, with associated equations, R^2^, and *p* values shown.

δ^15^N was negatively correlated with δ^13^C in western Taiwan and in eastern Taiwan in 2012–2014 (linear correlation, *p* < 0.05; Figure 4). There was no significant relationship (linear correlation, *p* > 0.05) between δ^15^N and δ^13^C values in eastern Taiwan in 2020 (Figure 4).

#### 3.3.2. Temporal Comparisons in Eastern Taiwan

Skipjack δ^15^N values ranged from 6.3 to 12.6‰ in 2012–2014 and from 7.4 to 12.3‰ in 2020. δ^13^C values ranged from −18.9 to −15.5‰ in 2012–2014 and from −18.7 to −17.0‰ in 2020. There were significant differences (*t*-test, *p* < 0.05) in mean (±SD) δ^15^N values between 2012–2014 (9.4 ± 1.4‰) and 2020 (9.8 ± 1.2‰). There were significant differences (*t*-test, *p* < 0.05) in mean (±SD) δ^13^C values between 2012–2014 (−17.7 ± 0.5‰) and 2020 (−17.9 ± 0.4‰).

Skipjack fork length was positively correlated with both δ^15^N and δ^13^C value in eastern Taiwan during both sampling periods (linear correlation, *p* < 0.05; Figure 3A,B).

Skipjack δ^15^N values were negatively correlated with δ^13^C in eastern Taiwan in 2012–2014 (linear correlation, *p* < 0.05; Figure 4), while the correlation between δ^15^N and δ^13^C was non-significant in eastern Taiwan in 2020 (linear correlation; *p* > 0.05; Figure 4).

δ^15^N values were negatively correlated with δ^13^C in western Taiwan and in eastern Taiwan 2012–2014 (linear correlation, *p* < 0.05; Figure 4), while the correlation between δ^15^N and δ^13^C was non-significant in eastern Taiwan in 2020 (linear correlation; *p* > 0.05; Figure 4).

**Figure 5:**
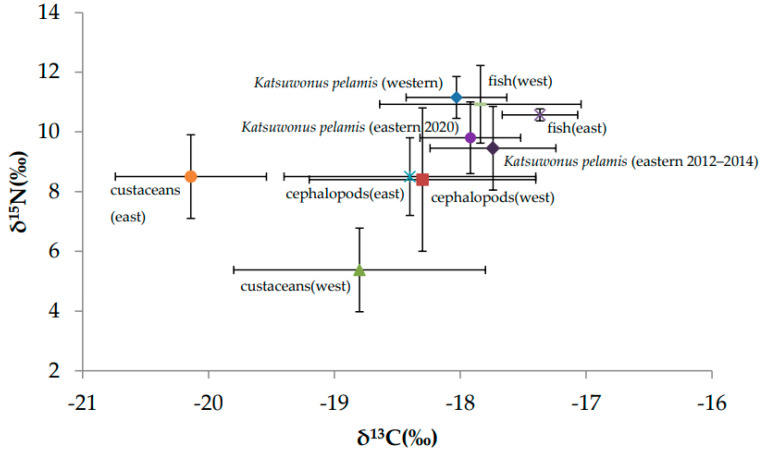
Stable isotope biplots of skipjack tuna and the prey in western and eastern Taiwan. Prey species were either collected and analyzed or taken from the literature [8,39,40].

A total of 45 prey samples were collected in western Taiwanese waters, with most prey represented by *Leiognathidae*, *Clupeidae*, *Caesionidae* spp., and *Trichiurus lepturus*. The prey SI data for TP estimates and mixing models were taken from six species for western Taiwan from [39], and included skinnycheek lanternfish, purpleback flying squid, mantis shrimp, shrimp postlarva, Amphipoda, and crab megalopa. (Figure 5). We divided all prey into three categories (fish, cephalopods, and crustaceans) for the subsequent Bayesian mixing model analysis. The prey SI data for TP estimates and mixing models were taken from nine species for eastern Taiwan from [8,39,40], and were represented by Cephalopoda, *Decapterus kurroides*, *Exocoetidae* spp., *Scombridae* spp., *Thysanoessa longipes*, *Thysanoessa inspinata*, *Nematoscelis difficilis*, *Euphausia pacifica*, and *Euphausia gibbooides*. (Figure 5). We divided all prey into three categories (fish, cephalopods, and crustaceans) for the subsequent Bayesian mixing model analysis.

### 3.4. Bayesian Mixing Models

#### Regional Comparisons

The most important prey from the Bayesian mixing models for skipjack in western Taiwan were cephalopods (63.3% for class I, 74.5% for class II, and 84.2% for class III, Table 6). The Bayesian mixing model results suggested cephalopods and crustaceans as the main prey of skipjack tuna in eastern Taiwan, though this varied by size class. The mixing models estimated that the most important prey for skipjack size class I were crustaceans (51.9%) and cephalopods (47.3%); size class II, cephalopods (53.5%) and crustaceans (42.4%); and size class III, crustaceans (56.8%) and cephalopods (37.9%) (Table 7).

## 4. Discussion

### 4.1. Diet Reconstructions from SCA and SIA

Skipjack tuna appeared to show generalist feeding on a wide variety of prey in the study regions. This is in accordance with past diet studies demonstrating fishes, cephalopods and crustaceans as important prey with variable relative proportions in different regions [47]; for example, skipjack diets were fish-dominated in regions of the North Atlantic but consisted equally of cephalopods and fishes in the eastern North Pacific [47]. Here, SCA showed forage fish as the main diet item both in western and eastern waters off Taiwan. The main prey item of skipjack tuna in western Taiwanese waters was *Leiognathus bindus*, a demersal, coastal species, while the main prey in 2012–2014 in eastern Taiwanese waters were the epipelagic *Myopsida* squid and *Decapterus* fish species, and in 2020 in eastern Taiwanese waters were epipelagic *Decapterus* and *Cheilopogon* fish species. This is likely linked to the Kuroshio Current, which flows past eastern Taiwan year-round and serves as a corridor for migratory predators and prey resulting in skipjack feeding on common local prey species. Collectively, these comparative results in western and eastern Taiwanese waters show that skipjack tuna likely adjust their feeding habits to available regional species, which varied both regionally and inter-annually.

Bayesian mixing model reconstructions of the time-integrated diet contrasted with the fish-dominated SCA results, suggesting the importance of cephalopods in western Taiwanese waters and cephalopods and crustaceans in eastern Taiwanese waters. SCA allowed for species-specific diet analysis, indicating the importance of different forage fish in western (*L. bindus*) and 2020 eastern (*Decapterus* spp.) Taiwanese waters. SCA limitations may have partially driven differences with mixing model results, as prey-specific digestion rates may partially confound SCA results [15]; prior work has shown that unidentifiable and inseparable digestive material can preclude identification of diet items using SCA [48]. As skipjack have a rapid digestive rate and can consume up to 15% of their body weight per day [49], imbalances of prey representation could have a particularly strong effect on SCA-based diet reconstructions. The frequency of empty stomachs in this study were high (51.16% and 68.4% in western and eastern Taiwanese waters, respectively), showing that high stomach evacuation during capture and/or rapid recent digestion of prey may also affect interpretations from SCA. High fish diets contrasted with SCA results from 2012–2014, which indicated cephalopods and crustaceans as the main prey of skipjack in that region. Bayesian mixing model represent longer term, isotopically-integrated diet, complementing the tendency of SCA to provide daily snapshots that are non-representative of the year-round feeding dynamics. Skipjack have been shown to utilize deep depths in other regions, feeding on common deep scattering layer organisms such as squids, crustaceans, and lanternfish. Mixing model results may indicate that when skipjack are outside the study regions they are more reliant on mesopelagic squids and crustaceans. This highlights the potential importance of the study regions off Taiwan for effective feeding on larger, more calorically rich fish prey.

### 4.2. Regional Effects on Diet

In this study, TP of skipjack suggested similar roles as mesopredators in both regions (TP = 4.5 off western Taiwan and TP = 3.9 off eastern Taiwan), with higher estimated trophic positions in western waters indicating a moderate but significant difference between regional trophic structure. Regional and ontogenetic changes in skipjack δ^15^N values may also be partially driven by regional oceanographic and biological factors. Intra-specific isotopic differences in other regions [50] have been shown to be linked to different productivity dynamics across spatial gradients. Consumer SI values have also been shown to vary across nearshore to offshore gradients [51,52], further demonstrating that nutrient abundance and overall productivity dynamics have regional effects on δ^15^N values and TPs of predatory fishes. The oligotrophic Kuroshio Current flows past eastern Taiwanese waters year-round, while the study region in western Taiwan is affected by three major ocean currents (i.e., China Coastal Current, Kuroshio Branch Current, and the South China Sea Surface Current), creating nutrient upwelling and fronts with nutrient-rich surface waters. The low nutrient current related to low primary production in the Kuroshio Current may result in longer pelagic food webs [38], and low N-fixation would lead to low baseline δ^15^N values [13]. Isotopic differences may also be driven in part by body size, as predator δ^15^N values have been shown to increase with body size [53] and samples from western Taiwan had a lower mean body size than in eastern waters. The relative influence of overall trophic structure and baseline isotopic dynamics, combined with size differences, could not be fully assessed here, but strong differences in nutrient availability between the study regions may explain the δ^15^N and TP differences of the skipjack tuna between the western and eastern regions, and the spatial and temporal variability of these regions could be further explored with SIA [54], including the influence of differing baselines [55]. SIA could also be used to study the impact of fisheries activities on the diet changes of commercially exploited species, as has been done elsewhere [56]. Skipjack tuna are likely important mediators in the transfer of energy from primary producers to apex predators, and by delineating ecological trophic structure, predator diet shifts across pelagic systems in response to anthropogenic factors (e.g., fishing pressure) may be better predicted and understood. The Kuroshio Current is a major global current system that is particularly crucial to North Pacific fisheries [57], and understanding the ecological shifts of pelagic predator foraging in response to changing biological conditions will improve future ecological models [58,59].

### 4.3. SI Dynamics across Skipjack Size

In general, δ^13^C and δ^15^N values increase with increasing body size in many marine forage fish and predator species [53,60] due to fractionation with each trophic step [52]. Prior work has shown diet changes with fish size due to the predator gape limitation [61] and changing foraging strategies [62]. Research in Eastern Australia also showed that prey size generally increased with increasing predator size [53,63]. The δ^13^C and δ^15^N values of skipjack tuna in eastern Taiwanese waters increased slightly but significantly with increasing body size due to successive trophic enrichment. In contrast, there was no significant relationship between body size and either δ^13^C or δ^15^N values in the western region, which is similar to patterns shown previously in estuarine systems in Taiwan [64] and in eastern Pacific Ocean pelagic systems [24]. The regional prey size and trophic structure may partially explain this trend, as size of available prey and trophic changes across prey ontogeny would drive the changing TPs of their consumers as larger prey become more common in skipjack diet.

Bayesian mixing models showed that as skipjack size increased in western Taiwanese waters, the dietaryproportion of *Clupeidae* and *Leiognathidae* slightly increased and *Caesionidae* decreased. However, there was no significant relationship between body size and either δ^13^C or δ^15^N values in the western region. This is likely due to the similarity of δ^13^C and δ^15^N values across forage fish, which would limit the isotopic indications of changes in dietary forage fish ratios. In contrast, in eastern Taiwan the increased proportion of teleosts (*Decapterus kurroides*, *Exocoetidae*, and *Scombridae* spp.) and decrease of cephalopods likely drove the positive correlation of skipjack size with both δ^15^N and δ^13^C. Prior feeding studies of juvenile skipjack showed that small prey include fish larvae and crustaceans (Euphausiacea, Amphipoda and Copepoda; [10]), but more fish and less crustaceans in skipjack in the Central Pacific [11] and decreasing proportions of fish and crustaceans and more cephalopods with increasing body size in the western North Atlantic [47]. Bayesian mixing model results suggest that in the regions studied here, skipjack tuna change their feeding habits with increasing body size, especially in western Taiwan, but not necessarily resulting in observable changes in SI values or estimated TPs.

Differences were observed across skipjack sizes, which differed between regions, but SI-inferred diet shifts were relatively slight. Short life spans and a relatively small change in size between small and large skipjack, compared to other pelagic teleosts of similar and higher TP, may also drive the observation of minimal change of TP across skipjack body size. Analysis of larger skipjack in western Taiwanese waters would support or refute the consistent TP across skipjack body size in that region. Overall, the Bayesian mixing models and SCA suggest that in regions off Taiwan, skipjack tuna change feeding habits with ontogeny, though these patterns will likely vary with shifting availability of local prey.

## 5. Conclusions

This study examined foraging patterns across time (eastern Taiwan; 2012–2014 and 2020) and space (eastern Taiwan versus western Taiwan) by skipjack in the Northwest Pacific Ocean. These data and results complement the recent applications of isotopic ecology to pelagic predators, including blue and black marlin, sailfish, yellowfin tuna, and Pacific bluefin tuna in Kuroshio Current waters. Complementary techniques (SIA and SCA) allowed for species-specific identification of diet items (using SCA) as well as time-integrated syntheses of trophic dynamics (using SIA). The results showed differences across regions and years, likely in response to local prey and oceanographic conditions. As such, local prey abundance likely determines skipjack trophic dynamics. Ongoing work combining tools to reconstruct trophic frameworks will help understand how the skipjack and other pelagic mesopredators will respond to shifting pelagic ecosystems.

## Figures and Tables

**Table 1:** δ^15^N and δ^13^C values for prey items of skipjack tuna used in the study.

Prey Item	*n*	Mean δ^15^N	Mean δ^13^C	Reference
Fish (west)	48	10.9 (±1.3)	−17.8 (±0.8)	This study, [39]
Cephalopods (west)	4	8.4 (±2.4)	−18.3 (±0.5)	[39]
Crustaceans (west)	17	5.4 (±1.4)	−18.8 (±1.0)	[39]
Fish (east)	14	10.6 (±0.2)	−17.4 (±0.3)	[8,39]
Cephalopods (east)	11	8.5 (±1.3)	−18.4 (±0.9)	[8]
Crustaceans (east)	198	8.5 (±1.4)	−20.1 (±0.6)	[40]

**Table 2:** Dietary composition for skipjack tuna (*Katsuwonus pelamis*) by frequency of occurrence (FO%), numerical abundance (N%), mean percentage of weight (W%), and index of relative importance (IRI) in western Taiwanese waters.

Prey Item	FO%	N%	W%	IRI
Teleostei				
Clupeidae				
*Spratelloides gracilis*	4.7	29.9	29.8	277.6
Myctophidae				
*Benthosema pterotum*	2.3	34.6	7.0	96.8
Leiognathidae				
*Leiognathus bindus*	39.5	29.9	53.1	3282.9
*Leiognathus rivulatus*	2.3	1.9	2.0	8.9
Caesionidae				
*Pterocaesio digramma*	4.7	2.8	5.8	39.9
Pomacentridae				
*Chromis fumea*	2.3	0.9	2.3	7.6

**Table 3:** Dietary composition for skipjack tuna (*Katsuwonus pelamis*) by frequency of occurrence (FO%), numerical abundance (N%), mean percentage of weight (W%), and index of relative importance (IRI) in eastern Taiwanese waters in 2012–2014.

Prey Item	FO%	N%	W%	IRI
Crustaceans				
*Sergestidae* spp.	2.5	1.2	0.1	3.4
*Euphausiidae* spp.	8.9	14.3	0.6	132.2
*Cymothoidae* spp.	8.2	1.7	0.4	16.9
Cephalopoda				
*Myopsida* spp.	25.3	12.5	37.5	1265.9
Zooplankton				
Stomatopoda	13.9	5.6	0.7	86.9
Amphipoda	1.3	2.2	0.1	2.8
Megalopa	5.1	1.9	0.1	10.0
Teleostei				
Exocoetidae				
*Exocoetidae* spp.	10.8	3.0	12.7	169.1
Carangidae				
*Decapterus macrosoma*	16.5	5.3	19.8	412.3
*Selar crumenophthalmus*	3.2	0.8	2.9	11.9
*Carangoides* spp.	1.9	0.5	0.2	1.4
*Decapterus kurroides*	1.9	0.6	2.9	6.7
Chaetodontidae				
*Chaetodontidae* spp.	1.3	0.3	0.1	0.5
Ammodytidae				
*Ammodytidae* spp.	10.1	26.5	7.8	347.7
Trichiuridae				
*Trichiurus lepturus*	5.7	1.2	0.9	12.2
Acanthuridae				
*Acanthuridae* spp.	1.3	0.2	0.1	0.4
Balistoidei				
*Ostraciidae* spp.	0.6	0.1	0.01	0.1
*Arothron stellatus*	0.6	0.5	0.1	0.4
*Monacanthidae* spp.	10.8	0.5	0.3	9.0
*Balistidae* spp.	3.2	1.1	2.6	11.8
Dactylopteridae				
*Dactylopteridae* spp.	1.3	0.4	0.1	0.7
Pterois				
*Pterois* spp.	9.5	0.2	0.03	2.3
Monacanthidae				
*Thamnaconus* spp.	0.6	0.1	0.1	0.1
Engraulidae				
*Engraulidae* spp.	5.7	19.2	9.8	165.1

**Table 4:** Dietary composition for skipjack tuna (*Katsuwonus pelamis*) by frequency of occurrence (FO%), numerical abundance (N%), mean percentage of weight (W%), and index of relative importance (IRI) in eastern Taiwanese waters in 2020.

Prey Item	FO%	N%	W%	IRI
Crustaceans				
Squillidae	6.7	14.2	1.8	111.1
*Sergestidae* spp.	1.8	1.5	0.1	2.9
Cephalopoda				
*Myopsida* spp.	17.6	10.2	23.1	442.7
Teleostei				
Clupeidae	2.4	13.7	0.6	34.5
Exocoetidae				
*Cheilopogon* spp.	16.4	14.9	26.3	609.0
Polymixiidae	1.2	0.6	0.04	0.3
Fistulariidae				
*Fistularia commersonii*	0.6	0.2	0.01	0.03
Carangidae				
*Decapterus* spp.	31.5	23.1	42.1	2054.3
Bramidae	4.2	1.5	0.1	2.9
Emmelichthyidae	0.6	0.2	0.02	0.1
Chaetodontidae				
*Chaetodon* spp.	0.6	0.2	0.2	0.2
Ammodytidae				
*Ariomma* spp.	1.8	9.5	1.1	19.2
Trichiuridae	6.1	4.4	1.8	37.5
Scombridae	6.1	2.3	15.3	106.7
Balistidae	1.8	1.1	0.1	2.0
Tetraodontidae				
*Lagocephalus* spp.	0.6	0.2	0.03	0.1

**Table 5:** δ^15^N and δ^13^C values and estimated trophic position of skipjack tuna (*Katsuwonus pelamis*) in western and eastern Taiwanese waters.

Location/Years	*n*	Fork Length (FL, cm)	δ^15^N Value (Mean ± SD)	δ^13^C Value (Mean ± SD)	Trophic Position
Western/2020	43	45.0 ± 4.2	11.2 (±0.7)	−18.0 (±0.4)	4.5
Eastern/2012–2014	149	49.7 ± 8.7	9.5 (±1.4)	−17.7 (±0.5)	3.8
Eastern/2020	167	54.1 ± 9.2	9.8 (±1.2)	−17.9 (±0.4)	3.9

**Table 6:** The proportion of prey families in skipjack tuna (*Katsuwonus pelamis*) in western Taiwanese waters, as estimated by Bayesian mixing models and isotopic composition of skipjack and prey.

	Class I (<39.9 cm, *n* = 4)			Class II (40–49.9 cm, *n* = 38)			Class III (>50 cm, *n* = 1)		
Prey Sample	Mean	5%	95%	Mean	5%	95%	Mean	5%	95%
Fish	13	0	58	18	0	75	11	0	73
Cephalopods	63	1	89	75	0	100	84	6	100
Crustaceans	23	11	44	7	0	29	5	0	24

**Table 7:** The proportion of prey samples in skipjack tuna (*Katsuwonus pelamis*) in eastern Taiwanese waters, as estimated by Bayesian mixing models and isotopic composition of skipjack and prey.

	Class I (<49.9 cm, *n* = 72)			Class II (50–59.9 cm, *n* = 41)			Class III (>60 cm, *n* = 54)		
Prey Sample	Mean	5%	95%	Mean	5%	95%	Mean	5%	95%
Fish	1	0	3	4	0	12	5	0	15
Cephalopods	47	8	100	54	18	99	38	0	84
Crustaceans	52	0	91	42	0	77	57	13	85

## Sample Availability

Samples of the compounds are not available from the authors.
